# On the Present State of Our Knowledge of Opium

**Published:** 1804-07-01

**Authors:** A. F. Gehlen

**Affiliations:** Berlin


### On the present State of our Knowledge of Opium.

*By* A. F. Gehlen, *of Berlin.*

Opium, a substance so long known, of such great importance in medicine, and such extensive practical utility, has been frequently examined, but by no means so thoroughly, as not to leave many doubts respecting its nature and origin. It is even not as yet ascertained, whether good opium be obtained by incision of the capsules of the poppy, as Mr. Kerr relates, in form of an extilled juice, or whether it be prepared by expressing the ripe poppy heads, by boiling them, and evaporating the juice. For the probability of the first method being employed in order to obtain this substance, it is alleged, that it contains albuminous matter, and that its aqueous extract is perfectly soluble in alcohol, which would not be the case, were it prepared in the latter way; no albuminous matter would then be found in it; on the contrary, a great quantity of mucilage, which the heads of the poppy, on being boiled, yield in a considerable proportion, and the presence of which must necessarily prevent the perfect solution of the aqueous extract in alcohol. However plausible these arguments may appear, they are not sufficient for removing all doubts, because if the proceeding in evaporating the expressed juice of the poppy heads were the same as that employed in our laboratories for the preparation of the inspissated juices of hyosciamus, cicuta, and other narcotic and acrimonious plants, a portion of albuminous matter will, notwithstanding, be contained in it. Against the perfect solution of the aqueous extract, as an argument of its containing no mucilage, it may be suggested, that the proximate constituents of vegetables, as long as they are mixed with one another, are acted upon in a quite different way if treated with them separately. Thus Mr. Buchholz found in 500 grains of select opium, 150 grains of gum, although the solution of the aqueous extract, mixed with an equal quantity of alcohol, appeared quite clear and transparent.

The interesting experiments of Cit. Dubuc render the preparation of opium, as related by Mr. Kerr, still more doubtful. He maintains, that at least the fourth part of opium consists of impurities, which, on examination, were found to be particles of the stem, leaves, capsules, and seeds of the poppy, most minutely divided; and it is to this admixture, that he ascribes the peculiar narcotic odour odour of that substance. He farther remarks, that this odour is very volatile, and often no more, or in a far less degree perceptible on the surface of opium, while it still remains strong and nauseous in the internal tough part; whence he concludes, that this volatile particle is merely accidental; an opinion which he endeavours to confirm by the following experiments.

1. On drying fresh and tough opium by a heat of 40—50° Reaumur till it becomes pulverisable, it entirely loses that stupifying odour, retaining that of purified opium or laudanum, from which it only differs by the heterogeneous particles which it contains. When these volatile particles, which are disengaged during the exposure of opium to the heat, are collected by a proper apparatus, they will for the most part be condensated to a clear liquor, the colour of which becomes a few days after straw yellow; one part of it, however, retains the form of gas, which is never perfectly miscible with water. Both give the odour of fresh opium, but in such a high degree, that animals brought into this mixture are immediately suffocated. Mr. Dubuc mentions here, that he has several times prepared an extract from the white poppy heads, at different times of their growth, without ever obtaining a substance of the true odour of opium, or laudanum; but he observed, that volatile-smelling particles were disengaged from a quantity of leaves of poppy, which being extremely similar to the substance obtained in the former experiment, induced him to make the following.

2. Leaves of the white poppy were squeezed and triturated in a stone mortar, by which they yielded a great quantity of a milky brownish juice which had a bitterish taste. On exposing it to the air, at a temperature of 10—12° Reaum. it had puffed in one day considerably, and gave at the same time a strong narcotic smell, similar to that which is exhaled by fresh opium. On the fourth day the exhalation had increased to such a degree, that no person could approach it without a violent head-ach. On the fifth day the vessel was exposed for about twelve hours to the action of the sun, and the juice frequently stirred. In a few hours the stupifying smell gradually diminished, and was followed by another much resembling that of azotic gas. The juice, together with the vegetable particles, acquired a darker colour, probably owing to the azote disengaging itself, which, united to the odorous principle arising from the mass, constitutes the above-mentioned peculiar smell.

 3. After

3. After having made this observation on the origin of the peculiar smell of opium, he took twelve pounds of poppy, nearly of their full growth, and treated them as in the foregoing experiment, but without exposing them to the sun. Two days after the whole mass began to ferment, and the narcotic odour to be disengaged, and two days after it became still more perceptible. He now expressed the juice, and having filtrated it, he evaporated it by a gentle heat to the consistency of an extract, but he did not succeed in obtaining true opium, because the juice, as it was thickening, gradually lost the narcotic smell, and only retained that of a scentless plant.

4. He was equally unsuccessful by inspissating the juice of poppy on flat vessels by the heat of the sun.

5. He gently evaporated the juice of the young heads of poppy, partly taken from the plant in flower, to half its quantity, in hopes, by the mass being more concentrated, of obtaining a substance retaining the odorous particles. The juice thus far inspissated, began to ferment somewhat later, than the former, and on the tenth day had received the above-mentioned smell; but on being evaporated, the extract showed no difference from that obtained by the foregoing experiments. Half of the juice was preserved for future experiments.

6. Thirteen ounces of poppy heads, full grown, but still green, and four ounces of leaves and of the stem, were squeezed and perfectly triturated into a thick glutinous mass. On being exposed to the contact of air, it soon passed into fermentation, and after four days it had the smell of opium. Part of it being evaporated at a temperature of about 40° R. retained a weak smell of laudanum, and much resembled common opium. Mr. Dubuc relates here some experiments he made for obtaining opium by making incisions into the peduncle and the inferior part of the capsules of poppy. A yellowish, almost scentless, but very bitter juice extilled, which received a darker colour by the contact of the air; its taste remained the same, but the smell soon became narcotic, and on being exposed to the sun it dried, retaining only the smell of laudanum. Mr. Dubuc observed two species of poppy, the heads of one of which were exactly globular, while the others were rather oblong. The former yielded opium without incisions, as it issued from two, three, or four sutures, that could be discovered on the peduncle; and after it had thickened, it received the colour and smell of laudanum. It could be collected in small pieces of about four grains each.

Mr.

Mr. Dubuc took two grains of this substance, which produced a long and quiet sleep.

7. He prepared the extract from a considerable quantity of poppy by decoction, which being saturated, became milky on cooling, without having the least smell of opium. He then evaporated it till it could be pulverised.

8. One part of this dry extract was mixed with as much of the juice preserved from the 5th experiment, as was sufficient to give it the consistency of common opium, and the whole mixture resembled laudanum in colour, smell, and taste.

9. The rest of the last mentioned extract was likewise mixed with as much of the residuum of the 6th experiment, as was required to give it the consistency of opium. Three days after, the preparation of this substance became so very similar to opium, that it might have been taken for good opium.

Although the results of these experiments are by no means decisive, yet it appears from them, that a similar method of preparing opium may be adopted in oriental countries, viz. by inspissating the expressed juice of the heads of poppy, and mixing it with the triturated and fermenting mass of the whole plant; because the opium extilling from the incisions of the capsules did not retain the strong narcotic smell of common opium. It is not unlikely that in preparing opium, the expressed juice is heated, and the green albuminous matter which is separated during that process, is exposed to the air, till it has received the narcotic smell, when it is mixed with the clear inspissated juice, formed into cakes, and wrapped in the leaves of the poppy. These experiments of Mr. Dubuc are confirmed by similar ones made by Mr. Kuehn, apothecary at Arnstadt.

The same uncertainty which takes place in the manner of preparing opium, also obtains with respect to our knowledge of its constituent particles. In the analysis of this substance, the different sorts ought to be attended to, and the results of their examination compared with each other, which would at the same time throw light on the whole composition of this substance, and account for the results of former analyses; particularly, if regard was had to the methods employed by different chemists in the chemical examination. The analysis which Mr. Buchholz has undertaken with opium is, in this respect, not quite satisfactory, though it is otherwise very accurate and excellent. The chemical examination of that substance, which has been been related by former chemists, may always serve to illustrate several points that are not yet well understood; on which account I shall here give the results of the proceedings undertaken by different chemists with opium, whereby the reader will be enabled to judge which points, in the knowledge of the chemical composition of that substance, requires still to be illustrated. The sensible quality which first attracts our notice on examining opium, consists in its narcotic smell. The constituent part which produces this smell is of a volatile nature, imparting itself both to water and alcohol, when these are distilled with opium. It seems not to possess the nature of essential oils; at least, Mr. Buchholz could not obtain any trace of it by distilling it with water; but the quantity with which he operated in his experiments consisted only of 500 grains, and was consequently too small to render his results quite certain. He administered two ounces of the concentrated distilled water of opium to a dog, on which it produced no deleterious effects; whence he concludes, that the efficacious parts of opium do not depend on those volatile particles, and that consequently opium may be boiled with its dissolvents without any fear of losing its efficacious particles. These conclusions, however, seem not to be sufficiently confirmed by that experiment, as it is known that dogs will bear a great dose of opium; especially, as the dog on which Mr. B. made his experiments was diseased, which certainly had some influence on the operation of the opium.

Mr. Dubuc mentions in one of his experiments, that an atmosphere impregnated with the exhalations of opium is deleterious to animals. It is, however, known that opium is not deprived of its efficacy by the loss of the narcotic smell, though it is not yet ascertained to which part of opium the vis narcotica adheres. Neumann and Hoffmann ascribe it to a particular constituent of opium; an opinion which has not much been regarded in modern times. This substance is said to be of an oily but not of a volatile nature, to rise in form of a fat, tough, and strongly smelling scum to the top of the liquor, if opium be disolved in water by infusion, digestion, or gentle decoction, and to possess the narcotic properties in a very high degree. Dogs that could take more than one drachm of opium, without any noxious effect, were killed by a few grains of that substance. From a pound of opium two or three drachms are said to be obtained. It communicates its smell and narcotic power to water distilled with it, but it is not volatilized.

tilized. Mr. Josse is of a different opinion on this subject. He maintains that the narcotic and excitant qualities of opium are contained in the glutinous matter of opium. In order to obtain this substance, he treated opium, divided into pieces of one ounce weight with water of 30—36° R. in the same manner as is done to obtain the gluten from the flower of wheat; by which proceeding the surface of opium was softened and covered with a gluey fibrous matter. After all the soluble parts were dissolved in water by continued kneading, the gluten remained as an elastic dark brown substance, of such a penetrating smell and taste of opium, that the smell of it alone would excite vomiting. Exposed to the air it dries up and loses its elasticity, resembling burned earth, but retains its strong smell of opium. If kept moist, it passes into putrefaction, spreading a great stench. Mr. Josse obtained from one pound of opium 6½ ounces of this substance, which, when dry, were about five ounces and some drachms. Exposed to dry distillation, it shews no difference from the glue of flour, or any other substance that contains azote. Digested with 4 parts of alcohol it imparts to the liquor a brown colour and a disagreeable smell; but the pieces of this substance used for digestion neither changed their form nor size, though they lost the eighth part of their weight, which, on being distilled, remains as a scentless very bitter resin, the alcohol itself passing over with the narcotic smell with which it was impregnated on being digested with opium. The above mentioned glutinous substance, thrown into hot oil, does not change its colour when it is dried; but when added to the oil in its fresh state it becomes green. It is said to be soluble in vinegar and other acid vegetable liquors, and to be precipitated by alkalis in flakes which have the smell of opium.

From these observations, Mr. Josse concludes, that opium is a preparation of the juice of poppy; that the acid corrigentia of opium are not capable of diminishing the much dreaded narcotic property, but, on the contrary, that the narcotic effect is increased by them, as being dissolvents of the glutinous substance; that Spanish wine is best adapted for dissolving the extractive parts of opium, as it does not act on the glutinous matter. Lastly, he adds, that from the common great poppy an extract may be prepared, resembling in its efficacy, as well as in other properties, true opium. It is, however, probable, that what Mr. Josse calls gluten, is nothing but the albuminous matter, which is separated by boiling from the expressed vegetable juices, as he compares it with a similar substance, contained in the extract. cicut. when prepared after Baron Stork's method.

[ To be continued. ]

